# Changes in Medical Schools

**Published:** 1856-08

**Authors:** 


*Changes in Medical Schools.*—We are not a little amused in noticing the paragraphs in the various journals, relative to the recent numerous changes in the organization of medical schools. The facts are stated — usually — in such a manner as to favor the personal interests of the locality in which the journal happens to be published. All this is fair enough in the present abundance of colleges and scarcity of students; but it is no reason why we should not laugh at it. We do not expect any gratuitous philanthrophy in the case, and perhaps it is too much in the present distresses of many schools to ask that the whole truth should be told.

The University of Louisville has lately lost two of its most distinguished teachers. This fact is freely announced in all the journals, and the insinuation conveyed that its fortunes are failing. Now if our good friends had chosen to go a little farther, and tell the whole truth, they would have said that the resignations of these two gentlemen were dictated by imperious personal reasons, and that the University of Louisville, always the great school of the west, is still unsurpassed in point of talent and high professional tone in its teachers.

Again, in all the journals whose notices of Prof. Flint's resignation we have perused, with the single exception of Reese's American Medical Gazette, it is simply announced that "Prof. Flint resumes his residence in Buffalo." This is interesting in view of the fact that he has never resided elsewhere, and still more interesting in view of the other fact that he resumes his connection with the University of Buffalo. The simple-minded reader would suppose that this latter was the more important announcement of the two, and would ask why it is that all these organs of medical news and purveyors of medical gossip, neglect to mention it. They all knew it — the fact was publicly announced three months ago — but they still forget it. They can recollect that Prof. Flint resigns at Louisville, but not that he accepts at Buffalo.

And why? It requires no divination to see that while the knowledge of the one fact among students may help Cincinnati, New York, or Philadelphia, the knowledge of the second will do no such thing and will help only Buffalo. All right, gentlemen! Do n't advertise unless you are paid for it

In the meantime we feel good-natured. While the star of other schools declines, we cannot expect them or their organs to do any gratuitous labor for Buffalo; but, *Deo volente*, we purpose to take care of that matter ourselves. We give below a list of all the changes that have occurred of late, east, west or south, with an occasional guess at probabilities for future organizations and appointments.

*Castleton Medical College, Vt.*—Prof. Geo. Hadley, who resigned the chair of Chemistry a year since, resumes it and lectures there during the fall term, closing in time to give his usual course in Buffalo.

*New York Medical College* (13th St. School.)—Prof. Edward H. Parker resigns the chair of Anatomy. No successor yet appointed.

*College of Physicians and Surgeons of New York.*—Prof. La Conte resigns the chair of Chemistry. It is rumored that Prof. St. John, of Cleveland, is to be appointed.

*Jefferson Medical College, of Philadelphia.*—Prof. T. D. Mutter resigns the chair of Surgery. Prof. Samuel D. Gross, of Louisville, is appointed to the vacancy.

*New Orleans School of Medicine.*—This is a new institution, headed by Dr. Fenner. It promises well, being made up of men of ability.

*University of Nashville.*—We see it stated that Dr. Robert M. Porter, Prof. of Anatomy in this institution, is dead. The vacancy thus occasioned will be eagerly sought for.

*University of Louisville.*—Prof. Austin Flint resigns the chair of Theory and Practice of Medicine. Prof. Lewis Rogers, so long the distinguished Professor of Materia Medica in the same school, succeeds him. Prof. Breckenridge is appointed to the vacant chair of Materia Medica.

Prof. S. D. Gross resigns the chair of Surgery, and Prof. Joshua B. Flint, of the Louisville Institute, succeeds him.

*Louisville Medical Institute.*—Prof. Joshua B. Flint resigns the chair of Surgery, and Dr. T. G. Richardson, formerly Demonstrator of Anatomy in the University of Louisville, succeeds him.

*Cleveland Medical College.*—Prof. Horace Ackley resigns the chair of Surgery. No successor appointed as yet. It is rumored, also, that Prof. St. John will resign the chair of Chemistry, to go to New York.

*University of Buffalo.*—The old faculty remains unchanged: the following additions are made. A new professorship of Clinical Medicine and Pathology is created, and Prof. Austin Flint appointed. Also a new professorship of Surgical Anatomy and Surgical Pathology, to which Prof. Edward M. Moore is appointed.

We believe this closes the list. One remarkable feature in these changes is that professorships formerly much sought for, now go begging. The reason for this is found in the fact that with few exceptions medical teaching does not pay particularly well.

